# Probiotics in Medicine: A Long Debate

**DOI:** 10.3389/fimmu.2020.02192

**Published:** 2020-09-25

**Authors:** Elisavet Stavropoulou, Eugenia Bezirtzoglou

**Affiliations:** ^1^CHUV (Centre Hospitalier Universitaire Vaudois), Lausanne, Switzerland; ^2^Department of Infectious Diseases, Central Institute, Valais Hospital, Sion, Switzerland; ^3^Laboratory of Hygiene and Environmental Protection, Department of Medicine, Democritus University of Thrace, Alexandroupolis, Greece

**Keywords:** probiotics, medicine, intestine, COVID-19, lung, allergy, *Lactobacillus*, *Bifidobacterium*

## Abstract

During the last years probiotics gained the attention of clinicians for their use in the prevention and treatment of multiple diseases. Probiotics main mechanisms of action include enhanced mucosal barrier function, direct antagonism with pathogens, inhibition of bacterial adherence and invasion capacity in the intestinal epithelium, boosting of the immune system and regulation of the central nervous system. It is accepted that there is a mutual communication between the gut microbiota and the liver, the so-called “microbiota-gut-liver axis” as well as a reciprocal communication between the intestinal microbiota and the central nervous system through the “microbiota-gut-brain axis.” Moreover, recently the “gut-lung axis” in bacterial and viral infections is considerably discussed for bacterial and viral infections, as the intestinal microbiota amplifies the alveolar macrophage activity having a protective role in the host defense against pneumonia. The importance of the normal human intestinal microbiota is recognized in the preservation of health. Disease states such as, infections, autoimmune conditions, allergy and other may occur when the intestinal balance is disturbed. Probiotics seem to be a promising approach to prevent and even reduce the symptoms of such clinical states as an adjuvant therapy by preserving the balance of the normal intestinal microbiota and improving the immune system. The present review states globally all different disorders in which probiotics can be given. To date, Stronger data in favor of their clinical use are provided in the prevention of gastrointestinal disorders, antibiotic-associated diarrhea, allergy and respiratory infections. We hereby discuss the role of probiotics in the reduction of the respiratory infection symptoms and we focus on the possibility to use them as an adjuvant to the therapeutic approach of the pandemic COVID-19. Nevertheless, it is accepted by the scientific community that more clinical studies should be undertaken in large samples of diseased populations so that the assessment of their therapeutic potential provide us with strong evidence for their efficacy and safety in clinical use.

Probiotics are living non-pathogenic microorganisms, which when given in sufficient amounts (at least 10^6^ viable CFU/g) should be beneficial to host by improving its microbial balance in gut and participate in the metabolism (1).

Moreover, probiotics are known to have particular properties such as; resistance to acid pH, bile tolerance, tolerance to pancreatic fluid, adhesion and invasion capacity in the intestinal epithelial cells (2). The above properties permit their survival in the gastrointestinal tract and the improvement of the intestinal balance (2).

During the past years, the use of probiotic microorganisms has been applied to modulate the microbiome in a beneficial way and thus fighting against infections threatening human and animal health (3). Their use might sometimes be an alternative to antibiotics permitting to reduce antimicrobial resistance due to the overuse or misuse of antibiotics against infections (4, 5). Spreading of antibiotic resistance is a major public health problem among human pathogens (4). The development of antibiotic resistance through different mechanisms may result in unsuccessful treatment of infectious diseases.

Nevertheless, neither the FDA, more the EFSA have approved the use of probiotics for preventing or treating health issues, despite their classification as safe food supplements (6, 7). Both authorities have punctuated the faulty characterization and health claims, the scarcity of an efficient explanation of their mechanism of action as well as the failing of considerable studies in humans to really show a benefit of the probiotics' administration.

The Japanese Ministry of Health and Welfare seems to have a different policy. FOSHU label (Food for Specified Health Use) is given to a specific probiotic product allowing health claims (8).

*Lactobacillus* and *Bifidobacterium* genera are principally reported as probiotics. These bacterial genera are isolated in the human intestine in considerable populations. *Lactobacillus* includes different species with the most semantic as probiotics; *L. acidophilus, L. rhamnosus, L. bulgaricus, L. reuteri, L. casei, L. johnsonii, L. pantarum*. These strains are acid-tolerant in the stomach acidity and have a good adherence capacity to the intestinal cells. *Bifidobacterium* belong to the phylum of Actinobacteria as they have a characteristic ramified morphology. The most common *Bifidobacterium* probiotic species are *B. animalis, B. bifidum, B. breve, B. infantis, B. lactis, B. longum*.

*Streptococcus thermophilus, Enterococcus faecalis, Enterococcus faecium, Pediococcus, and* several Bacilli, as well as the yeasts *Saccharomyces boulardii* and *Saccharomyces cerevisiae* also show some probiotic properties.

Probiotic microorganisms are part of our intestinal flora, yet they can also be found in other ecological environments. However, it must be clear that probiotic properties are strain-related and even tissue-dependent. Thus, the probiotic effect neither universal to all bacterial species nor to all human tissues.

Early intestinal colonization seems to provide protection against certain diseases by strengthening our immune system (1). Until now, it is not known which bacterial species are necessary to induce an appropriate and effective “barrier effect” against pathogens, but it seems that this “barrier effect” can be strongly supported by providing beneficial food supplements called probiotics (3). Specifically, through this type of diet a beneficial microbiota dominated by Lactobacilli (*phylum* of Firmicutes) and Bifidobacteria (*phylum* of Actinobacteria) is registered (9).

International Scientific Association for Probiotics and Prebiotics (2014) (8) declared that metabolic by-products, bacterial molecular components and dead microorganisms might have some beneficial effect; despite the real conviction that a probiotic must have a high ability to survive under intestinal conditions (acidic pH, enzymes, bile salts, etc.) and that their activity and effectiveness are linked to viability (10). Currently, the term “postbiotic” is developed for soluble bacterial components with biological activities which are believed to be safer than the use of whole bacteria (11).

Multiple studies on the probiotic strains' characteristics have been done, including biochemical profile, adherence and invasion capacities to intestinal cells (12). Moreover, pharmacokinetic studies (half - life time, intestinal permeability, correlation of the obtained dose and persistence in stools) have been reported (13) as well as studies on the tolerance of the probiotic strain by the host and its input on the bacterial microflora (14). All the above tests and studies have permitted to characterize by the Food and Drug Administration (FDA) (USA) a probiotic given strain under the acronym of “GRAS” (Generally Recognized As Safe), meaning a food supplement which is considered safe by experts (6).

The bacterial colonization of newborns by vaginal delivery or cesarean section has been thoroughly studied in relation to the immune system, the diet, the environment as well as many other involved factors (1). Hospital staff, personal habits, infections, stress, hormonal status, vaccination and agedness seem to be crucial factors for the establishment of the bacterial microflora which is actually called “microbiome” (1).

New technological applications have been brought into light. Next generation sequencing (NGS) methodologies include sequencing of the 16S ribosomal RNA gene (r RNA) as well as metagenomic sequencing. Without any doubt technological developments have empowered scientists with advanced knowledge and stand for an in depth perception of the human ecological communities of commensal, symbiotic and pathogenic microorganisms. Microbes are able to participate in multiple metabolic processes in our intestine, synthetizing and producing key nutrients as well as repulsing pathogenic bacteria. To this end, the “Human Microbiome Project” in the United States (US National Institutes of Health, NIH, http://commonfund.nih.gov/hmp/) (15) and the metaHIT Consortium (https://www.gutmicrobiotaforhealth.com/metahit/) in Europe (16) have been able to shed light on the composition of bacterial populations in detail at the various human sites.

Without any doubt, diet is the major player of the microbiota composition. Diet consists of introducing chemical substances following consumption preferences in our intestinal ecosystem in different timing (17, 18). On another hand, host physiology, immunological status and metabolism capacity regulate the response to the bacterial colonization and the presence of specific microbial species (1).

The Gut-associated lymphoid tissue (GALT) system sits in the intestinal wall furnished by immunological elements able to protect the intestinal wall from invasion. Malfunction of the GALT following treatment by antibiotics, inappropriate alimentation or stress leads to dysbiosis and increasing of the intestinal permeability (1, 19). GALT malfunction results in an impaired immunity, either inefficient or exacerbated. Therefore, infectious diseases as well as immune-mediated diseases (20, 21) such as allergy (22) and auto-immune (inflammatory) disorders (23–25) may occurred following disruption of the equilibrium between microbiota and host, the so-called dysbiosis. Along these lines, probiotics seem to be beneficial in these issues as they stimulate the host immune system and preserve the microbial intestinal balance *via* the barrier effect (1).

Probiotics seem to exert their effect through different mechanisms (1, 20) (Figure 1);

- **Competition for space** (Spatial arrangement theory) in the intestinal lumen and wall (26, 27).

- **Antagonism** between pathogenic bacteria and probiotics which is produced by competition for nutrients found in limited quantities in the intestine (26) or by pH modulation. Maintenance of an acid pH on the epithelium by probiotics (27, 28).

- **Synthesis of nutrients** reported as sources for energy for epithelial cells or bacteria (26).

- **Maintenance of mucosal integrity**. The intestinal epithelium is part of the intestinal mucosa layer. This epithelium is monolayer and the locus in between the epithelial cells is tightly unified by transmembranar proteins. The intestinal mucosa has 2 more layers, the *lamina propria* and the *muscularis mucosae*, which bracket the epithelial monolayer. Probiotics show a cytoprotective action upon the gastric mucosa integrity by strengthening the epithelial junctions and preserving the mucosal barrier function (29).

**-Enhance intestinal barrier function**. Preservation of the microbial intestinal balance *via* the barrier effect (1).

- **Regulation of gut motility** (30). Intestinal motility as well as reflexes and secretory functions of the gastrointestinal tract are regulated by the Enteric Nervous System (ENS) found in the intestinal wall. ENS is considered as a second brain as it is composed of a complex neural network of sensory, motor, inter neurons and glial cells. In this vein, there is a reciprocal communication between Central Nervous System (CNS) and bacterial flora in the intestine. The CNS affects the microbiota by altering the motility and permeability of the gut or even *via* mediators secreted by neuro-endocrine cells (30).

- **Prevention of osteoporosis**. Studies showed that probiotic supplementation can both increase bone density and protect against primary (estrogen-deficiency) and secondary osteoporosis (31).

- **Hypocholestaemic action** (32). Hypocholestaemic effect is bacterial species related. Different mechanisms have been proposed. Deconjugation of bile acids (30), assimilation of endogenous or exogenous cholesterol (33), binding of cholesterol and free bile acids to the microbial cell (34) or co-precipitation of the free bile acids (34).

- **Anti-carcinogenic, antimutagenic and anti-allergic activities** (35–37). Studies in animals as well as cohort studies in humans have demonstrated a correlation between consumption of dairy products and the risk of colorectal cancer. Anticancer activity of some strains is associated to the capacity of the probiotic strain to inhibit or reduce DNA destruction in the very first stages of carcinogenesis.

- **Production of H**_**2**_**O**_**2**_ by probiotics promotes epithelial restitution (38).

- **Production of antimicrobial agents, organic acids and bacteriocins** (39–41) stimulates the production of intestinal mucins which will prevent the implantation of pathogens.

- **Their action on the intestinal immune system** (1, 42) by stimulating the receptors of innate immunity, TLRs which will cause the production of pro-inflammatory cytokines and lead to the initiation of phagocytosis by macrophages.

**Figure 1:**
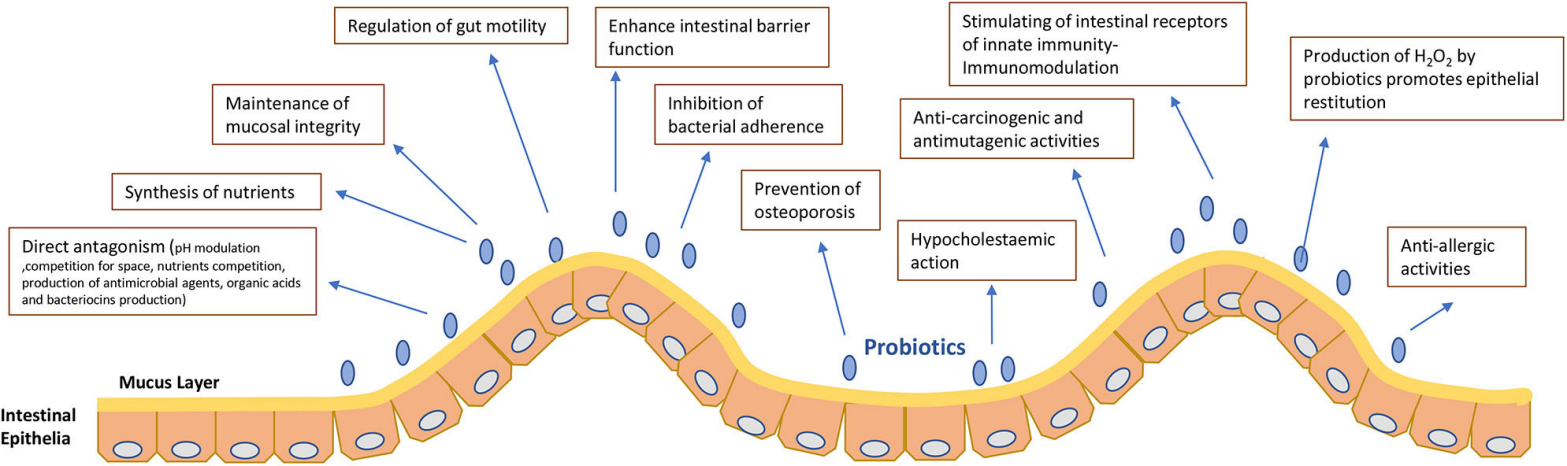
Mechanisms of action and properties of probiotics.

Specifically, some probiotics induce activation of CD4 + and CD8 + T lymphocytes and secretion of IgA by *lamina propria* plasma cells to neutralize pathogens, while other probiotics suppress the Th1 inflammatory response and the production of inflammatory cytokines IL-12, TNF-a, and stimulate Treg lymphocytes (39). Therefore, they appear to have a multifaceted role as an adjuvant to immunity or as a stifle of inflammatory responses.

Undoubtedly antibiotics play a crucial role in the treatment of infections, however their uncontrollable use has led to serious ecological consequences but also for human health, as for example the increase of multi-resistant strains and the alteration of human floras and specifically of the intestinal microbiota and its functions (5). In this vein, probiotics have been proposed as a “non-invasive” alternative therapy or co-therapy to antibiotics. As already stated, probiotics help to preserve the normal human microbiota in a beneficial status.

According to WHO, probiotics are “living microorganisms, which, when consumed in adequate amounts, have a health benefit for the host.” Historically, Metchnikoff was the first scientist to propose the use of probiotics. They may be used as food supplements to consume or as medicines in the form of pills or powder containing a single or a combination of several strains. It is generally accepted that the dose of probiotic microorganisms must be 100 million to 10 billion microorganisms for it to be effective.

## Gastrointestinal Diseases

Without any doubt, the strongest evidence supporting the use of probiotics is related to the treatment of gastrointestinal diseases and specifically the acute diarrhea.

### Gastroenteritis

Probiotics are of scientific interest for intestinal pathology. Their effects are reported in multiple clinical studies (Table 1).

**Table 1:** Use of probiotics in gastrointestinal disorders.

**Disease state**	**Probiotic**	**References**
Gastroenteritis	*-Lactobacillus*	(43)
	*-Lactobacillus F19 and L. reuteri*	(44, 45)
	*-L. casei (431strain)*	(45, 46)
	*-S. thermophilus* and *B. bifidum (*TH-4+ BB12 strain)	(46, 47)
	*-L. acidophilus* (NCFM strain) + *B. lactis* (Bi-07 strain)	(48)
Antibiotic associated diarrhea and traveler's diarrhea	*-Lactobacillus GG, E. faecium* (SF68 strain) and *S. boulardii*	(49, 50)
	- *Saccharomyces cerevisiae* variant *boulardii* CNCM I-1079	(51, 52)
	*-Lactobacillus* Rosell-52, *Bifidobacterium* Rosell-175 and *Lactobacillus* Rosell-11	(51, 52)
	-*L. reuteri* DSM 17938 *and L acidophilus* LB (low efficacy)	(53)
*Clostridioides difficile* infection	*-S. boulardii, Lactobacillus* spp.	(54–58)
Inflammatory bowel disease	*-Escherichia coli* Nissle 1917 or *Lactobacillus* GG	(59–63)
	*-Bifidobacterium* spp. and *L. acidophilus*	(59–63)
	*-S. boulardii*	(64)
	*-Lactobacillus GG and L. johnsonii*	(65)
Celiac disease	*-Bifidobacterium* spp. and *L. acidophilus, various*	(59–63, 66)
*Helicobacter pylori* infection	*-Bifidobacterium* BB-12	(67)
	*-L. acidophilus* La-5	(13)
Lactose Intolerance	*-Lactobacillus delbrueckii subspecies bulgaricus* and *S. thermophilus*	(68)

Some strains of *E. coli*, as well as *Salmonella* spp*., Shigella* spp*., Campylobacter* spp. *and* viruses like Rotavirus, Norovirus etc are among the most frequent causes of gastroenteritis leading to inflammation of the intestine. *Lactobacillus* strains are the main players of the commercially available probiotics. *Lactobacillus* strains have been shown to be effective against the pathogens *E. coli and C. difficile* (43).

Specifically, *Lactobacillus* F19 and *L. reuteri have* a positive impact on the gastrointestinal microbiota by boosting the immunological status (44, 45). Similarly, *Lactobacillus casei* (431strain) is boosting the immune response and contributes on the faster convalescence of the diarrheal disease in children (45, 46). *Streptococcus thermophilus* and *Bifidobacterium bifidum (*TH-4+ BB12 strain) drop the risk of the *Rotavirus* diarrhea and colic's in children (46, 47). *Lactobacillus acidophilus* (NCFM strain) + *Bifidobacterium lactis* (Bi-07 strain) have a beneficial effect on the gastrointestinal ecosystem by reducing abdominal floating (48).

### Antibiotic Associated Diarrhea and Traveler's Diarrhea

*Lactobacillus GG, Enterococcus faecium* (SF68 strain), and *Saccharomyces boulardii* have a strong recommendation for the prevention of antibiotic-associated diarrhea, the treatment of *C. difficile* colitis, as well as treatment of gastroenteritis in addition to oral rehydration therapy (49, 50) (Table 1). *Saccharomyces cerevisiae* variant *boulardii* CNCM I-1079 has been given successfully to treat and prevent acute cases of antibiotic-associated diarrhea and traveler's diarrhea as described (51, 52). The same authors (51, 52) observed that *Lactobacillus* Rosell-52, *Bifidobacterium* Rosell-175 and *Lactobacillus* Rosell-11 strains were able to prevent pathogen invasion and treat traveler's diarrhea.

Nevertheless, studies have shown that a *Lactobacillus acidophilus* commercial strain acquired vancomycin resistance of *vanA* genes from enterococci in the gastrointestinal tract of germ-free mice *in vitro* and in *vivo* (69).

Similarly, we should note that the SF68 strain of *Enterococcus faecium* is a possible recipient of the van A gene cluster (70). Thus, *Enterococcus faecium* SF68 strain profile has led to concerns about the safety of probiotics containing this strain (70).

Those studies underpin the issue of the potential health risk when consuming probiotic foods containing enterococci which could be potential recipient of glycopeptide resistance genes (70).

Following EFSA and the Panel on Additives and Products or Substances used in Animal Feed (FEEDAP) (2008), Microbial Inhibition Concentration(MIC) antimicrobials standards have been designated for most common probiotics*; Lactobacillus, Streptococcus thermophilus, Pediococcus, Lactococcus, Leuconostoc, Enterococcus, Bifidobacterium, Propionibacterium*, and *Bacillus*.

More studies bring into light that the glycopeptide resistance of *Lactobacillus* strains is different from Enterococci and underline the safety of *Lactobacillus* strains used as probiotics with regard to their vancomycin resistance (71).

*L. reuteri* DSM 17938 *and L. acidophilus* LB have a lower recommendation (53). In a Cochrane's study (72), the efficacy of probiotics on acute infectious diarrhea in subjects of all ages was studied and a reduction in the duration of diarrhea by 1 day was demonstrated. There is no doubt that the evidence is weak and the methodological limits are questionable.

### *Clostridioides difficile* (Formerly Clostridium) Infection Pseudomembranous Colitis

As aforementioned, probiotics can be given as part of the treatment of *Clostridium difficile* colitis (Table 1). We focus our interest in this part during the last years; we notice an increase of *C. difficile* cases worldwide, associated to the misuse of antibiotics (54). The effect of administering probiotics on a *Clostridium difficile* has been evaluated in several studies with questionable results (73). A Swiss study finds that the action of probiotics depends on the basic risk of patients (>5%) of developing post-antibiotic pseudomembranous colitis (55, 74, 75).

*C. difficile* infection is a cause of post-antibiotic nosocomial diarrhea, the so called pseudomembranous colitis. The infection is due to gut microbiota alterations as a result of antibiotic treatment or other causes of microbiome alterations such as travel (76).

*Clostridioides difficile* and its spores are ubiquitous in the environment and thus are able to colonize the human intestine (74). The intestinal mucus appeared to be an important chemotactic factor for colon colonization by *Clostridium difficile* (55). Recently, the epidemics observed of *Clostridium difficile* seem to be linked to extremely virulent clones of this anaerobic bacterium in several countries; the genotypes 027 and 078 (77, 78). The infection could induce a toxic megacolon and lead to intestinal perforation and septic shock (73). In Switzerland, the disease is not obligatory reportable and only the clone 078 has been isolated (74, 75).

In a European global study on hospital strains, the ribotype 078 was the 11th most frequent ribotype in Europe. Of all 14 European countries enrolled in this study, Greece represented >10% of the strains with the virulent ribotype 078 (54). Moreover, in Greece, the hypervirulent genotype 027 has not been found, whereas genotypes 017 and 126 predominate (79). In Spain, the genotypes 078 and 126 predominate in patients (80). Globally in Southern Europe (Greece, Italy, Portugal and Spain), the genotypes 078 and 126 and 017 are the most common dominating genotypes (77). The genotype 017 is also found in Bulgaria and Poland (79).

It is of note that most patients having *Clostridium difficile* infection have been exposed to longterm antibiotics suffer from comorbidities and are elderly. It seems that the prevalence of the disease is closely associated to the abuse of antibiotics, specifically fluoroquinolones, cephalosporins as well as penicillins (78). Yet, most of the cases are hospital acquired (64%) compared to the community acquired cases (79).

The European Society of Clinical Microbiology and Infection (ESCMID) (2009) issued treatment guidance for *Clostridium difficile* infection (73), reviewing different treatment protocols such as antibiotics, immunotherapy, toxin-binding resins and polymers, probiotics, and fecal or bacterial intestinal transplantation. Guidelines are specified following disease state. Recommended antibiotics, are mostly vancomycin and fidaxomicin and less metronidazole than it used to be. In cases of recurrences fecal transplantation is recommended in combination with oral antibiotic treatment. Probiotics (*Saccharomyces boulardii, Lactobacillus* spp.) are also given in combination with oral antibiotic treatment (54, 56–58).

Probiotics (*Saccharomyces boulardii, Lactobacillus* spp*.)* can also be given in combination with oral antibiotic treatment (55).

Although several studies showed a moderate evidence on the beneficial effect of probiotic prophylaxis in *C. difficile* diarrhea (56, 57), a Cochrane study analysis suggests to adjunct them to the antibiotic therapy in the treatment of CDI (58). However, a study on the use of *Saccharomyces boulardii* in immunocompromised patients showed occurrence of invasive disease (81, 82). Another study on probiotic use as prophylaxis agent showed increased mortality due to non-occlusive mesenteric ischemia (83).

Therefore, the ESCMID guidelines do not recommend probiotics as an adjunctive treatment for CDI.

In consent with the European guidelines, the Infectious Diseases Society of America (IDSA) together with the Society for Healthcare Epidemiology of America (SHEA) issued similar guidelines summarizing that due to lack of evidence probiotics are not recommended (84).

### Inflammatory Bowel Disease

Probiotics seem to have an effect on inflammatory bowel disease (85, 86) (Table 1). Studies on colitis animal models show a decrease in inflammatory status and the expression of inflammatory mediators.

Administration studies of the various probiotics remain differentiated in terms of treatment effectiveness. While some authors report that there is no difference in the relapse rate but simply a longer interval without relapse for the treatment of ulcerative colitis with probiotics such as *Escherichia coli* Nissle 1917 or *Lactobacillus* GG, others accept that supplementation of medical treatment with *Bifidobacterium* spp. and *L. acidophilus* improves clinical response (59–63). In addition, remission seems to be observed in pediatric subjects after long-term probiotic treatment as a supplement to medical treatment with mesalazine (87). Administration of *S. boulardii* in combination with mesalazine shows a considerable positive effect. Probiotics have also been applied in the treatment of Crohn's disease (64). Fewer recurrences were observed after administration of *Lactobacillus* GG and *Lactobacillus johnsonii* (65).

Pouchitis is the inflammation in the lining of a pouch as a result of colorectal surgery. In this context, some researchers have used probiotics for the treatment of active pouchitis inducing remission in 69% of subjects (65, 88), while other authors doubt the efficacy of probiotics on the treatment of pouchitis (66).

### Celiac Disease

To date, it is well known that gluten is the trigger in celiac disease. Nevertheless, it is suggested that intestinal microbiota might play a certain role in the pathogenesis and progression of the disease (66). Dysbiosis in the microbiota of patients with celiac disease has been reported in some studies with an intestinal microflora characterized by an abundance of *Bacteroides* spp. and a decrease in *Bifidobacterium* spp. (66). This could suggest that probiotics might have a beneficial effect on this condition (Table 1).

### Other Intestinal Associated Pathology

Intake of *Bifidobacterium* BB-12 has an impact on *Helicobacter pylori* infection in humans (67). Likewise, *L. acidophilus* La-5 also impacts *Helicobacter pylori*, boosts the immune effect and alleviate diarrheal symptoms (13) (Table 1).

### Treatment of Lactose Intolerance

Lactose intolerance is due to the inability to digest lactose in dairy products. It is believed to affect 60% of the world's population (89). However, lactose malabsorption varies considerably in the different countries from 5 to 15% in Northern Europe and America to 50–100% in South America, Asia and Africa. *Lactobacillus delbrueckii subspecies bulgaricus* and *S. thermophilus* in yogurts improve the intolerance to lactose as they possess the enzyme beta- galactosidase (68) (Table 1). Recently, randomized double-blind studies showed the efficiency of probiotic bacteria in fermented and unfermented milk preparations given to alleviate the clinical symptoms of lactose malabsorption (89).

## Allergy

Allergy results from an exacerbated hypersensitivity response of the immune system to usually harmless triggering substances in the environment. These substances are called allergens and they usually include drugs, foods, grass and tree pollen, insects, insect's bites and stings, dust mites, pet dander, chemicals and latex.

This hypersensitivity in immune system tolerance mechanisms seems to be modulated by the gut microbiota (90). Dysbiosis has been incriminated for the development of allergies (91). Probiotics have been successfully used in the treatment of allergic diseases such as allergic rhinitis, asthma, atopic dermatitis and food allergy (90). However, there is still controversy over their use.

Two meta-analyses reported improvement in the prevention of atopic dermatitis by the use of probiotics (92, 93). *Lactobacillus* alone and *Lactobacillus* along with *Bifidobacterium* seem to be protective against the development of atopic dermatitis, specifically when given early in pre- and postnatal high allergy risk populations as well as in the general population (93) (Table 2).

**Table 2:** Use of probiotics in allergy.

**Disease state**	**Probiotic**	**References**
Atopic dermatitis	-*Lactobacillus*	(93)
	*-Lactobacillus + Bifidobacterium*	(93)
	*-L. paracasei*	(94, 95)
	*-L. salivarius (LS01)*	(94, 95)
	*-L. fermentum*	(94, 95)
Allergic rhinitis	*-L. casei, L. rhamnosus, L. johnsonii EM1, L. acidophilus, L. gasseri, L. paracasei*	(96–99)
	*- B. lactis NCC2818 (Nestle)*	(100)
	*-L. paracasei (LP-33 strain)*	(101)
	*-Lactobacillus GG (LGG) + L. gasseri, L. acidophilus+ Bifidobacterium lactis*	(102–104)
	*- VSL#3 (4 Lactobacillus+3 Bifidobacterium+ 1 Streptococcus thermophiles)*	(102–104)
	*-Lactobacillus+Bifidobacterium*	(105)
	*-L. casei*	(106)
Atopic eczema	*-Bifidobacterium BB-12*	(90, 107, 108)
	*-B. longum, B. clausii,E. coli Nissle (EcN) 1917*	(90, 107, 108)

*Lactobacillus paracasei, Lactobacillus salivarius* (LS01 strain), and *Lactobacillus fermentum* are also used for the treatment of atopic dermatitis as antiallergic in children (94, 95). The use of probiotics in allergic conditions such as atopic dermatitis is promising (109).

Initially treatment was succeeded with a single probiotic strain. Among *Lactobacillus* species the most common in the therapy of allergic rhinitis are*; Lactobacillus casei, Lactobacillus rhamnosus, Lactobacillus johnsonii EM1, Lactobacillus acidophilus, Lactobacillus gasseri, Lactobacillus paracasei* (96–99).

As stated previously, *Bifidobacterium* BB-12 alleviate symptomatology of the atopic eczema.

*Bifidobacterium longum, Bacillus clausii*, and *Escherichia coli* Nissle (EcN) 1917 are also used as pharmatherapeutics (107, 108).

The use of *Bifidobacterium lactis* NCC2818 (Nestle) as well as *Lactobacillus paracasei* (LP-33 strain) seems to reduce the severity of the symptoms of allergic rhinitis (100, 101).

Recently, combination treatment was made possible with more than one probiotic strains. *Lactobacillus GG (LGG)* + *L. gasseri, L. acidophilus* + *Bifidobacterium lactis and also the commercial mixture VSL#3 consisting of 4 lactobacilli*+*3 bifidobacteria*+ *1 Streptococcus thermophilus* were used combined (102–104).

The combination of *Lactobacillus* + *Bifidobacterium* in treatment seems to be the most popular and successful for the allergic rhinitis (105).

*Lactobacillus casei* administered in children with mite allergies decreased the frequency and severity of symptoms (106).

Studies on the role of probiotics in prevention or treatment of food allergies were conducted but with contradictory results (90, 110). Heterogeneity of strains, duration and dosage of treatment should probably explain some of the differences.

## Respiratory Diseases

### Asthma

A raise in asthma and respiratory diseases has been observed during the last years in most industrialized countries (111).

As formerly discussed, the gut microbiota plays a capital role in the development of allergic diseases.

The modulation of the normal gut microbiota in an experimental model of asthma in animals has been registered (112). Children at risk of asthma showed microbial dysbiosis in their intestine with complete absence of certain bacterial genera (112). Hence, the administration of these “missing bacteria” in models of mice showed a decline in respiratory tract inflammation indicating their potential role as a causal agent in asthma (112). Recently, there appeared evidence that *Enterococcus faecalis FK-23* suppresses the asthmatic hypersensibility which seems to be associated with attenuation of Th17 cell development (113) (Table 3).

**Table 3:** Use of probiotics in respiratory diseases.

**Disease state**	**Probiotic**	**References**
Asthma	*Enterococcus faecalis FK-23*	(113)
Cystic fibrosis	-Various	(114–118)
Respiratory infections (global)	*L. rhamnosus* GG	(119)
	*L. reuteri* DSM 17938	(120)
	*L. reuteri* ATCC 55730	(121)

However, meta-analyses and double-blind randomized controlled studies did not perceive any substantial benefit from probiotic treatments (122–124).

### Cystic Fibrosis

Cystic fibrosis is an autosomal recessive disorder caused by a mutation in the CF transmembrane conductance regulator (CFTR) gene encoding the CFTR protein which regulates the movement of chloride and sodium ions across epithelial cell membranes. The result is a defective ion transport with a buildup of thick mucus throughout the body, leading to respiratory insufficiency, along with many other systemic disorders, mostly digestive. Moreover, the decreased mucociliary clearance in the respiratory tract, combined to the defective ion transport allows the proliferation of *Pseudomonas aeruginosa* as well as other pathogens in the respiratory tract which become more and more resistant due to iterative treatments and result in a repetitive inflammatory response (114).

Other than chronic respiratory disease, cystic fibrosis is associated, as aforementioned with digestive disorders (pancreatic, biliary and intestinal) also resulting in chronic inflammation as well as malabsorption of nutritious substances.

Probiotics seem to be promising for certain respiratory tract diseases including cystic fibrosis (115) (Table 3). In the case of cystic fibrosis there is a dysbiosis and frequent antibiotic therapy is able to unbalance the microbiota (116). In this context, the use of probiotics has been studied as it appears that they may reduce the rate of pulmonary exacerbations in the disease (117, 118).

### Respiratory Infections and COVID-19

*L. rhamnosus* GG reduces the risk of respiratory and gastrointestinal infections in infants (119).

Viral respiratory infections affect morbidity and mortality of a population. Pattern recognition receptors (PRRs) are the main sensor players of the innate immune response. Expression of many Pattern Recognition Receptors (RRs) is exacerbated in the lung cells during inflammation. In this vein, macrophages, monocytes, neutrophils are responding by increasing levels of PAMPs (Pathogen-Associated Molecular patterns) and DAMPs (Danger-Associated Molecular Patterns) (125).

It is known that intruder's pathogens have a specific unique profile of PAMPs resulting in a specific immune response (126). PAMPs are necessary molecules for the pathogens survival; hence they are not produced by the host. In viruses, the major PAMPs are nucleic acids or glycoproteins. PAMPs should be recognized by PRRs conducting to the expression of cytokines, chemokines, and other co-stimulatory molecules in order to eliminate the pathogenic virus and activating then antigen presenting cells and specific adaptive immunity (127, 128). The most studied PRRs for pathogens recognition are TLRs (Toll Like Receptors) which are membrane glycoproteins (129).

Without any doubt, there is a clear diversity in the patterns for pathogen recognition and host protection upon viral infections.

On the other side, PRRs recognize DAMPs (Danger-Associated Molecular Patterns) as danger signals released by damaged or necrotic host cells which reinforce the pro-inflammatory response (130). As a result, stimulation of TLRs is crucial for the protection from the development of diseases.

Probiotics' regulatory effect on the expression of Toll-like receptors (TLRs) was observed in several disease cases (131). Specifically, probiotics impede or reduce inflammation by minimizing the expression of TLR4 (132). In this vein, a large randomized controlled trial was conducted (PROSPECT Investigators and the Canadian Critical Care Trials Group) for the use of probiotics in critically ill patients of intensive care units (ICU) with ventilator-associated pneumonia (VAP) showing beneficial and salutary effects of probiotics in this seriously ill patients (133).

Although, it has long been known that Coronaviruses cause respiratory and sometimes gastrointestinal diseases with mostly like clinical presentations, the SARS-CoV-2 has recently monopolized our interest due to the COVID-19 pandemic as a result of its contagiousness as well as unexpected mortality rates.

Coronaviruses are recognizably different than most enveloped viruses in nature as they are localized in the lumen of the ERGIC (ER-Golgi intermediate compartment). The ERGIC mediates between the endoplasmic reticulum and the Golgi on the secretory pathway for releasing of the infectious virions from the infected cell (134). This is where the majority of the E protein is localized, participating in the assembly and budding of the infectious virion.

It is known that virus replication in the host for establishment of an infection occurs through host-viral PPIs (Protein-protein interactions) (135, 136). In SARS-CoV-1, The E protein has only been mentioned to connect to five host proteins (Bcl-xL, PALS1, syntenin, sodium/potassium (Na^+^/K^+^) ATPase α-1 subunit, and stomatin) (135).

Latest information states that Coronaviruses encode PBM-containing proteins that bind to cellular PDZ proteins. Those proteins keep an important role in the anchoring receptor proteins (135). Alongside, E protein interaction partners are identified as p38 mitogen-activated protein kinase (MAPK) inhibitors. Clearly, this is an important therapeutic tool as studies have shown that inhibitors of p38 mitogen-activated protein kinase (MAPK) prolonged survival in mice (137, 138).

*Lactobacillus* contains a HSP27-inducible polyphosphate (poly P) fraction. Probiotic-deriving polyphosphates have the ability to strengthen the epithelial barrier function and keep intestinal homeostasis through the integrin-p38 MAPK pathway (139).

As most treatments targeting Coronaviruses are currently ineffective, bringing into light more interaction partners for Coronavirus protein E could enhance a therapeutic approach.

*L. reuteri* DSM 17938 showed beneficial effects against upper respiratory tract and gastrointestinal symptomatology (120).

Similarly, *L. reuteri* ATCC 55730 has been shown to alleviate respiratory tract and gastrointestinal symptomatology in workers in Sweden (121).

During the last years, the gut–lung axis in bacterial and viral infections is considerably discussed (140). It seems that intestinal microbiota amplifies the alveolar macrophage function (140) and as a result, gut microbiota acts as a protective mediator during pneumonia (141).

We state here the importance to clarify the involved mechanisms of the probiotic efficiency in respiratory diseases which could potentialize their use as prophylactic or field therapy.

In conclusion, more knowledge is necessary to define the role of probiotics as therapeutics in viral and other respiratory diseases, as we have as yet only scratched the tip of this complicated issue.

## Neurological and Psychiatric Diseases

During the last years, there is increasing interest in the use of probiotics for prevention and treatment of neurologic diseases (Table 4). Recent studies stated the potential role for microbiota in the pathogenesis of neurological and brain disorders (150–152).

**Table 4:** Use of probiotics in neurological and psychiatric diseases.

**Disease state**	**Probiotic**	**References**
Neurological and psychiatric diseases	-*L. rhamnosus*	(142–144)
Autism Spectrum Disorder (ASD)	-*L. acidophilus* Rosell-11 -Various	(145–147)
Autoimmune myasthenia gravis	Various	(148)
Autoimmune encephalomyelitis	Various	(149)

It is believed that there is reciprocal communication between the central nervous system and the intestine, the so-called “microbiota-gut-brain axis” (150, 152). which is a model of interaction between the intestinal microflora and the brain.

Physical and psychological stress may interfere in the control of the intestinal and the vaginal microflora. Higher numbers of the putrefactive bacteria *Clostridium sp* are found under stress (153, 154).

Early-life events such as stress, environmental factors or other may impact the intestinal microbiota (155).

Studies report that the ingestion of *Lactobacillus rhamnosus* regulated the transcription of γ-aminobutyric acid (GABA) receptors and therefore emotional behavior (154). The intestinal microbiota affect GABA which transmit signals to the brain trough enteric nerve s (142).

Normal microbiota preserves the intestinal balance by improving epithelial tight junctions reducing gut permeability (150). Otherwise, in case of cellular damage, multiple immune and inflammatory responses are produced as well as activation of the spinal neurons and the *vagus* nerve (156). As a result, inflammatory cytokines are produced affecting the central nervous system (156).

Autism spectrum disorder (ASD) is a developmental disability that can cause problematic behavior in social, emotional, and communication skills. It seems that there is a connection between gut bacteria and autism (145, 146).

Taking the above into account, we understand the pivotal role of the gut microbiota in the neural and brain development and regulation and general and mental health affection in case of imbalance. Maternal feeding plays an important role in gut microbiota of the newborn (1), as it contains probiotics.

Studies showed potential effects of probiotics in the treatment of neurologic diseases (143) (Table 4).

Positive effect of probiotics have been observed in the progression of the autoimmune myasthenia gravis (148), the autoimmune encephalomyelitis (149), as well as on motor behaviors (144). Moreover, probiotics seem to have neuroprotective properties (157) and a positive impact on cognition (158).

Improvement of the antisocial behavior, communication and concentration problems was observed in a cohort study of children with ASD treated with the probiotic strain *L. acidophilus* Rosell-11 during a period of 2 months (147).

Probiotics have been studied in the treatment of the above neurological diseases. However, the current evidence on their efficacy is poor. As already discussed, their efficiency is strain, tissue, and dose dependent.

Studies undertaken are not uniform in terms of the chosen characteristics of the population, the population size, the undertaken therapies and procedures and the suitable strain or strains' mixture.

Rare cases of adverse effects of probiotics have been observed (159). It is also notable to state that immaturity of the neurological system, mental retardation and other severe neurodevelopmental problems predispose those patients at potentially higher risk of adverse effects.

Without any doubt, when the mechanisms of action of the probiotics are completely understood, we would then be able to evaluate their safety and efficiency issues on neurological diseases (140).

## Liver Diseases and Hepatic Encephalopathy

There is mutual communication between the central nervous system and the liver, the so-called “microbiota-gut-liver axis.” Thereby, there is a reciprocal interaction between hepatic receptors (Toll-like receptors) and bacterial lipopolysaccharides. When intestinal imbalance occurs prompting the alteration of the intestinal permeability, immune and inflammatory responses are produced and can result in hepatic disorders. Moreover, nutrients absorbed by the gut reach the liver.

Cirrhosis seems to be associated with changes in the presence of *Bifidobacterium* species in the intestinal microbiota (160).

Moreover, modifications of the intestinal microbiota have been observed among patients with chronic hepatitis B (161), primary sclerosing cholangitis (160) and proliferation of hepatocellular carcinoma (161). There was diversity observed in intestinal microbiota amongst subjects with hepatitis B virus-related cirrhosis and subjects with chronic hepatitis B (160, 161).

Hepatic encephalopathy is closely related to the intestinal microbiota (162). The metabolic activity of the intestinal microbiota on amino-acids results in the production of toxic substances (NH3, phenols, amines, phenolic acids) that are inactivated in the liver. However, when liver failure occurs these substances are not inactivated, they enter the circulation, cross the hematoencephalic barrier and cause hepatic encephalopathy resulting in coma.

The mainstay of treatment of hepatic encephalopathy is lactulose and lactitol, laxative agents that can be considered as prebiotics as they lower blood ammonia concentrations, possibly by favoring colonization with acid-resistant, non-urease producing bacteria. Moreover, they also act by altering the colonic pH, improving gastrointestinal transit and increasing fecal nitrogen excretion (163).

In this same spirit, probiotics have also been proposed as an adjunctive treatment for hepatic encephalopathy.

Lactic acid bacilli, more specifically *Lactobacilli* and *Bifidobacteria* seem to be the most effective species for hepatic encephalopathy but *Clostridium butyricum, Escherichia coli* Nissle 1917, *Streptococcus salivarius*, and *Saccharomyces boulardii* are also used (164) (Table 5).

**Table 5:** Use of probiotics in Liver diseases.

**Disease state**	**Probiotic**	**References**
Cirrhosis	*Bifidobacterium* sp.	(160)
Hepatic encephalopathy	*-Bifidobacterium* sp*., Lactobacillus* sp*., C. butyricum, E. coli Nissle 1917, S. salivarius, S. boulardii*	(164)
	*- C. butyricum, B. infantis*	(162, 165)

Treatment with the probiotics *Clostridium butyricum* and *Bifidobacterium infantis* seems to be promising as an adjuvant therapy for the management of the mild hepatic encephalopathy (162, 165).

According to a meta-analysis of 21 trials, when compared to placebo/no treatment, probiotics seem to be beneficial in treatment of hepatic encephalopathy but do not seem superior compared to lactulose (165).

Other than the treatment of hepatic encephalopathy, probiotics have also been studied as prophylaxis to prevent recurrence with beneficial effects (166).

Based on the fact that changes in gut microbiota are inducing liver diseases, an increasing interest in regulating the gut microbiota by probiotics for the treatment of liver disorders is reported (167). However, the current evidence on this efficacy is unclear. As stated previously, the efficiency of a probiotic is strictly strain, tissue, and dose dependent.

It is necessary to proceed to high-quality randomized clinical trials with standardized procedures on the undertaken probiotic therapy with appropriate strains and diligent choice of the population size and characteristics in order to get knowledge on the efficacy and safety of probiotics. In these terms, probiotics cannot be recommended for the treatment of most hepatic disorders, aside from some implication on hepatic encephalopathy.

## Genito-Urinary Infections

Bacterial vaginosis is a vaginal inflammation caused by an imbalance of the vaginal flora with overgrowth of several bacterial species and decrease in Lactobacilli. It seems that women at reproductive age develop more frequently vaginosis.

Historically, Albert and Döderlein in 1892 stated the importance of the vaginal flora in women's overall health (168).

*Lactobacillus* spp. and specifically the species *L. crispatus* are predominant in the bacterial flora of healthy women (169). As known, *Lactobacillus spp* produce lactic acid protecting the vagina from colonization by pathogens. However, when the balance is disturbed and *Lactobacillus* are decreasing or missing vaginosis occurs.

In this vein, scientists have tried to restore this imbalance of the vaginal flora by oral or vaginal administration of lactobacilli (170, 171) (Table 6).

**Table 6:** Use of probiotics in Genito-Urinary tract infections.

**Disease state**	**Probiotic**	**References**
Bacterial vaginosis	*-Lactobacillus* spp.	(169)
	*-L. crispatus*	(169)
	*-L. acidophilus*	(170, 171)
	*-L. rhamnosus GR-1*	(170, 171)
	*-L. fermentum RC-14*	(170, 171)
*Gardnerella vaginalis*	*-Lactobacillus* spp.	(172)
	*-L. acidophilus*	(172)
Urinary tract infections (UTIs)	*-Lactobacillus rhamnosus GR-1*	(173)
	*-L. reuteri RC-14*	(173)
	*-L. casei Shirota*	(173)
	*-L. crispatus CTV-05*	(173–175)
	*-L. rhamnosus GG*	(173)

*L. acidophilus* seems to have a positive effect in prevention and treatment of bacterial vaginosis (171, 176).

However, the mechanism of action of *Lactobacillus* remains unclear. While some scientists claim that the production of lactic acid inhibits the establishment of pathogens by creating an acid environment (1), *in vitro* studies have stated that the production of H_2_O_2_ or bacteriocins by certain *Lactobacillus* protects against pathogens involved in bacterial vaginosis (176, 177). Thus, the adherence of *Gardnerella vaginalis* to the vaginal epithelium is inhibited by *Lactobacillus* strains (172). Studies have shown that the bacteriostatic effect of *L. acidophilus* on *G. vaginalis* NCTC 11292 dropped by 60% when culture pH turned to alkaline by the addition of NaOH or by catalase denaturation of the H_2_O_2_ (172) (Table 6).

Moreover, it seems that administration of *L. acidophilus* or *Lactobacillus rhamnosus* GR-1 and *Lactobacillus fermentum* RC-14 for an extended period of 2 months is beneficial in the vaginosis treatment (170, 171).

In conclusion, probiotics can be effective in preventing and treating vaginal imbalance in bacterial vaginosis. Studies undertaken are encouraging. More research and clinical studies are necessary in order to define whether or not probiotics are efficient for the prevention and treatment of vaginosis and which strains should be involved.

Urinary tract infections (UTIs) are among the most common infections. Shortness of the women urethra is associated with more frequent urinary tract infections in women. They are divided in uncomplicated and complicated and include cystitis, pyelonephritis, febrile UTIs, prostatitis and urinary-source bacteriemia. UTIs mostly result when uropathogens, mostly from the fecal flora, ascend the urinary tract or from seeding of the kidneys *via* bacteremia or following medical interventions (urinary catheters, urological surgery). Clinical presentations vary from asymptomatic bacteriuria to urethritis, cystitis, prostatitis, pyelonephritis and bacteriemia.

UTIs are associated with an important economic impact due to many hospitalisations as well as morbidity, mortality and most importantly development of microbial resistance (178–180).

Development of multi-drug resistance has led doctors to look for milder prophylactic therapies in order to minimize the high cost of therapies (181).

Probiotic effects on urinary tract infections remain controversial (181–183) (Table 6). While a team states that there is no benefit from probiotics administration (182), other scientists observe a shortening of the average duration of illness, as well as a considerable reduction of the infection rate (181, 184).

An extended review based on a search of PubMed for relevant articles showed the efficacy and safety of probiotics as prophylaxis against potential pathogenic bacteria of the urinary tract (185). *Lactobacillus rhamnosus* GR-1 and *L. reuteri* RC-14 seemed to be the most effective probiotic strains followed by *L. casei* Shirota and *L. crispatus* CTV-05 (185). The effectivity of *L. crispatus* in UTIs has been observed by many authors (186, 187).

On the contrary, *L. rhamnosus* GG is not shown to be sufficiently effective (185). It seems that probiotics' activity and efficiency is closely related to the specific administered strain. Hence, all authors agree on the safety issue of their use (185).

## Metabolic Syndrome and Cardiovascular Diseases

### Diabetes

The gut microbiome seems to play a role in the development of diabetes. Studies in animals have shown that some species of *Lactobacillus* and *Bifidobacterium* could prevent or reduce the severity of type 2 diabetes (188) (Table 7).

**Table 7:** Use of probiotics in metabolic syndrome and cardiovascular diseases.

**Disease state**	**Probiotic**	**References**
Diabetes	*-Lactobacillus*	(188)
	*-Bifidobacterium*	(188)
	*-*Various	(189–191)
Obesity	-Various	(175)
Cardiovascular disease and cholesterol	*-Lactobacillus*	(41, 188–192)
	*-Bifidobacterium*	(41, 188–192)
	*-*Various	(41, 183, 188–190, 192)

Studies in humans have attempted to assess the factors that justify metabolic changes, oxidative stress and inflammation (188).

Lactic acid bacteria show antioxidant activity. Diabetic subjects are characterized by constant systematic inflammation with high levels of proinflammatory cytokines [TNF-a, IL-6, b kinase inhibitor (IKKb), and Jun N-terminal kinase (JNK)] which exert a negative effect on insulin. Lactic acid bacteria have positive clinical effects on the treatment of specific populations with type 2 diabetes by modulating the inflammatory status.

A meta-analysis of 12 randomized controlled trials showed that probiotics considerably improved the fasting plasma glucose (FPG) and the glycosylated hemoglobin (HbA1c) in type 2 diabetes (189). Similarly, the role of probiotic supplementation in persons developing type 2 Diabetes Mellitus (T2DM) in improving glycemic control showed an overall beneficial effect (190).

Our body is exposed to different physico-chemical or pathological conditions having as outcomes the production of free radicals (ROS) which causes the oxidation in the human cell. Hence, it has developed endogenous antioxidant mechanisms to keep the homeostasis. Oxidative stress is occurring when imbalance between free radicals (ROS) and antioxidant mechanisms are taking place (193). In the case of diabetes, disorders in lipid peroxidation, enzymatic systems as well as impaired glutathione metabolism are observed.

Pathogenesis of diabetes is then characterized by a potent oxidative stress, as increased levels of reactive oxygen species (ROS) are present (194).

The consumption of yogurt with live probiotics seems to touch up the antioxidant status and fasting plasma glucose (FPG) levels in type 2 diabetic patients (191).

## Obesity

The significance of the human gastrointestinal microbiota is stated in the case of obesity as well.

Obesity is linked to structural and functional changes in the gastrointestinal ecosystem. The intestinal microbiota of obese patients is characterized by increased numbers of bacteria of the phylum of *Bacteroidetes*. Low numbers of bacteria of the phylum of *Firmicutes* are observed.

Diversity and abundance of certain bacterial populations can trigger metabolic pathways leading to obesity (173).

Obesity is an important risk factor for type 2 Diabetes mellitus and cardiovascular diseases due to the high levels of inflammation mediators which (39) are recorded in obese persons. Administration of probiotics and antibiotics - has been used to stimulate weight gain in farm animals (3, 40). However, there is controversy in their efficacy by authors (174).

In humans, probiotics supplementation seems to decrease values of metabolic parameters and leads to the reducing of the weight gain in obese adults (175) (Table 7).

*Lactobacillus* spp. are associated with weight gain in children treated for diarrhea (195). Without any doubt more research is needed for estimating the role of probiotics in obesity.

## Cardiovascular Disease and Cholesterol

Cardiovascular diseases are a pivotal cause of death in the western world.

Multiple studies in animal and humans have demonstrated an important correlation between cholesterol levels and the risk of coronary heart disease. Dietary interventions suggest lowering of fat (low-saturated-fat diets) for the prevention of cardiovascular disease.

Supplementation of diet with fermented dairy products containing lactic acid bacteria seems to be in lowering blood cholesterol alleviating the cardiovascular disease (Table 7).

In this context, *Bifidobacterium* spp. and *Lactobacillus* spp. have been studied as food supplements with very promising effects.

However, the mechanisms of action of the antihypercholesterolemic potential of probiotics are yet under study. Several scientists propose a cellular pattern, which includes the binding of cholesterol to cellular surfaces and/or cholesterol assimilation by growing cells and cholesterol incorporation into the cellular membrane (196). Others rather accept a chemical pattern which comprises the deconjugation of bile via bile salt hydrolase, the coprecipitation of cholesterol with deconjugated bile (192) or the production of short-chain fatty acids by oligosaccharides (41).

## Cancer

Gastrointestinal (GI) cancers are a major health problem, accounting for 20% of all cancers and 9% of all causes of cancer death in the world (197).

The role of the human intestinal microflora has been extensively discussed in cancer disorders. Microbiota imbalance seems to be linked to cancer. One of the most representative examples is the correlation between *S. bovis/S. equinus* complex infection and intestinal cancer, principally colorectal (198).

Functional foods and probiotics seem to have a protective role against cancer development (Table 8) and also reduce the incidence of the post-operative inflammations (206).

**Table 8:** Use of probiotics in cancer and cancer cellular lines.

**Disease state**	**Probiotic**	**References**
Tumor cell apoptosis	*-L. casei*	(199)
	*-B. longum*	(199)
	*-L. acidophilus*	(199)
Inhibition of human colon cancer cell lines including HT-29, SW 480, Caco-2	*B. adolescentis SPM0212*	(200)
Anti-proliferative and pro-apoptotic effects in human gastric cancer cells and colonic cancer cells	*-L. paracasei IMPC2.1*	(201)
	*- L. rhamnosus GG*	(201)
	*-L. acidophilus 606*	(202)
	*- LGG/Bifidobacteriumanimalis* subsp. *lactis*	(203)
Antitumor activities	*-Bacillus polyfermenticus*	(204)
	*-L. acidophilus NCFB 1748*	(205)

As shown by some investigators, probiotics possess anti-proliferative and pro-apoptotic properties against gastrointestinal cancers (207, 208) (Table 8).

Similarly, milk fermented with *Lactobacillus casei, Bifidobacterium longum*, and *L. acidophilus* showed beneficial effects on tumor cell apoptosis (199).

Moreover, in cellular lines it has been observed that *Bifidobacterium adolescentis* SPM0212 inhibited the proliferation of three human colon cancer cell lines including HT-29, SW 480, and Caco-2 (200) (Table 8).

Anti-proliferative and pro-apoptotic effects of *Lactobacillus paracasei* IMPC2.1 and *L. rhamnosus GG* strain have been observed in both human gastric cancer cells and colonic cancer cells (201) (Table 8).

Furthermore, antitumor activities have been shown by *Bacillus polyfermenticus* (204).

In addition, *L. acidophilus* 606 (202), and LGG/*Bifidobacterium animalis* subsp. *lactis* (203) have also shown activity against human colon cancer cells.

*L. casei* Shirota is reported to prevent intestinal dysbiosis and having an effect on bladder cancer by lowering fecal enzyme activity (205). Lowering of fecal enzyme activity has also been observed by *L. acidophilus* NCFB 1748 which decreases cancer risk and radiotherapy-related diarrhea (205).

Nevertheless, most of the above studies were performed *in vitro*, in cellular lines or in animal models displaying the efficiency of probiotics in gastrointestinal cancers. As stated previously, those studies ascribe in probiotics properties such as, anti-carcinogenic effect, anti-mutagenic effect, derangement in differentiation process in tumor cells, modifications of tumor gene-expressions, inhibition of pro-carcinogenic bacteria and improvement of the immune system and intestinal balance (206).

However, the mechanisms inducing these effects are not completely understood. It is of paramount interest to expand these studies in humans with specific clinical trials which could strengthen our knowledge of the potential probiotic strain related efficiency and safety, as well as of the administration procedure and dosage for the different types and stages of cancer (206).

## Osteoporosis

Human and animal studies indicated that probiotic supplementation may be a therapeutic tool to the prevention and treatment of bone loss as they strengthen bones and skeleton (31). Moreover, probiotics protect against primary estrogen-deficiency and secondary osteoporosis (31) (Table 9).

**Table 9:** Use of probiotics in osteoporosis.

**Disease state**	**Probiotic**	**References**
Osteoporosis	-Various	(31)

## Oral Diseases

Gingivitis is an inflammation confined to the gingiva, while in periodontitis the inflammation process affects all peridental tissues and the alveolar bone.

*P. gingivalis, A. actinomycetemcomitans, T. forsythia, Staphylococcus intermedius, Candida albicans*, and *T. denticola* are the main pathogens associated to periodontitis.

*Lactobacillus salivarius* WB21 modulates the oral microbiota and reduces risk of gingivitis and periodontitis (209) (Table 10). Chewing gums or lozenges with probiotics can improve periodontal disease (214).

**Table 10:** Use of probiotics in oral diseases.

**Disease state**	**Probiotic**	**References**
Gingivitis	*-L. salivarius* WB21	(209)
Periodontitis	*-L. salivarius* WB21	(209)
Dental caries	*-L. reuteri*	(210)
	-Various	(211)
Halitosis	-Various	(212)
Oral candidiasis	*-L. rhamnosus GG*	(213)

Dental caries is tooth decay due to acids made by bacteria. Following excess of sucrose, *Streptococcus mutans* found in the oral cavity are able to adhere to the teeth enamel causing demineralization of the tooth enamel. Probiotics can reduce levels of *Streptococcus mutans*. It is reported that systematic yogurt and fermented by *Lactobacillus reuteri* bovine milk consumption for 2 weeks reduces *Streptococcus mutans* population in the oral cavity by up to 80%(210) (Table 10). Moreover, fluid or lozenges containing probiotics reduce *Streptococcus mutans* levels (211).

Halitosis (Chronic bad breathing) is the unpleasant breath odor attributed to the production of volatile sulfur compounds especially during the mornings due to poor oral hygiene or dental caries, periodontitis and other oral conditions. Reduction of the volatile sulfur compounds is observed by H_2_O_2_ produced by probiotics. Additionally, probiotics compete for colonization in the oral cavity (212) (Table 10).

Lastly, oral candidiasis (oral trush) is an opportunistic mycosis, due to colonization of the mucous membranes of the oral cavity mostly by *C. albicans* on the mucous membranes of the oral cavity. Candidiasis occurs when the normal oral microbiota balance is disturbed in immunocomprised patients, elderly or patients receiving long antibiotic regimens. *L. rhamnosus* GG seems to reduce the prevalence of *C. albicans* which is a normal component of our oral microbiota (213) (Table 10).

## Autoimmune Diseases

Dysbiosis is linked to the pathogenesis of Sjogren's autoimmune syndrome (215) as a possible interaction between the human microbiome and the clinical manifestations of the disease seems to exist. All the same, if the human microbiome is revealed to play a key role in the pathogenesis of Sjogren's disease (215), the next step could be new and promising therapeutic approaches such as the administration of probiotics as an adjunctive treatment to immunosuppressive therapy (Table 11).

**Table 11:** Use of probiotics in autoimmune diseases.

**Disease state**	**Probiotic**	**References**
Sjogren's syndrome	-Various	(215)
Rheumatoid arthritis	-Various	(23, 216)
Systemic lupus erythematosus	-Various	(23, 216)
Multiple sclerosis	-Various	(23, 216)

Randomized controlled trials have shown promising results in modulating beneficially human microbiota in autoimmune diseases such as rheumatoid arthritis, systemic lupus erythematosus and multiple sclerosis (23, 216) (Table 11).

## Vaccine Adjuvant

*Lactobacillus casei* 431 was used for boosting the immune response and *Lactobacillus fermentum* strain VRI 003 (PCC) as an adjuvant to flu vaccine and athletic endurance (217, 218) (Table 12).

**Table 12:** Use of probiotics as vaccine adjuvant.

**Disease state**	**Probiotic**	**References**
Vaccine adjuvant	*Lactobacillus casei* 431	(217, 218)
Adjuvant to flu vaccine	*Lactobacillus fermentum* strain VRI 003 (PCC)	(217–219)

Probiotics were shown effective in increasing immunogenicity levels by auctioning upon seroconversion and seroprotection rates in adults inoculated with Influenza vaccines (219) (Table 12).

## Discussion

The normal microbiota of the intestine plays a very important role in the body's defenses (1) and in the occurrence of multiple diseases such as, infections, autoimmune and allergic diseases and other (218–222). Probiotics seem to be a modern approach to prevent and reduce the symptoms of these diseases or as an adjuvant therapy by maintaining the proper balance of our intestinal microbiota (114).

A plethora of commercial products with differences in strain(s) composition and potentiality have been developed as probiotics and functional foods; Align *B. infantis* (4 mg/capsule $\frac{1}{4}$ 1 billion CFU), Activia yogurt (*B. lactis*; 100 million bacteria per gr), Culturelle (*L. rhamnosus* GG (*L. rhamnosus*:10 billion bacteria plus insulin 200 mg per capsule), Culturelle for kids (*L. rhamnosus*:1.5 billion bacteria per packet), Florajen *(L. acidophilus*: 20 billion bacteria per capsule), Florastor *S. boulardii* lyo: 250 mg per capsule), Howaru (*L. acidophilus/B. lactis*: 10 billion bacteria per capsule), Kefir (*L. lactis. L. rhamnosus, L. plantarum, L. casei, L. acidophilus, L. reuteri, Leuconostoc cremoris, Streptococcus diacetylactis, S. florentinus, B. longum, B. breve, B. lactis*: 7–10 billion CFU per cup), Lactinex (*L. acidophilus, L. bulgaricus*: 106 CFU/tablet and 109 CFU/packet), RepHresh Pro-B (*L. rhamnosus, L. reuteri*: 5 billion CFU per capsule; vaginal use),VSL#3 *(L. acidophilus, L. plantarum, L. paracasei, L. bulgaricus, B. breve, B. infantis, B. longum, S. thermophilus*: 225 billion bacteria per 2 capsules), Yakult *(L. casei*: 8 billion bacteria per 80 mL bottle), Ultralevure (*Saccharomyces boulardii* CNCM I-745: 250,00 mg, 50 mg/cap) (223).

Adverse effects (224) of probiotics have been reported such as abdominal cramps, loose stools, bloating, gas and flatulence.

Moreover, as probiotics are “living organisms,” their use is not recommended in immunocompromised patients, such as those receiving corticosteroid therapy and other immunosuppressive treatment, transplanted and oncological patients especially those undergoing chemotherapy. Besides, probiotic treatment is not recommended in patients with prosthetic valves as there have been reports of infective endocarditis due to probiotics (225).

It is also important to have a deep understanding of the role of CYP enzymes (226). CYP enzymes represent a superfamily of enzymes that play an important role in the process of activating or inactivating a plethora of therapeutic agents. The high metabolic rate of the gut microbiome is due to the many enzymes that catalyze reactions in the metabolism of drugs. This high enzymatic activity of the intestinal microflora is linked to the presence of P450 in the main bacterial strains from the human fecal microbiota. The fact that many intestinal bacterial strains have CYP enzymes (P450), arises the question that if living probiotics express P450 activity it could possibly influence the metabolism of drugs and their bioavailability? (226).

In addition, if the intestinal barrier is disrupted (226–228), this metabolism is affected. Undoubtedly, the role of probiotics is to restore deficiencies and imbalance in the intestinal microbiome and to establish a protective effect. Albeit that, the high enzymatic activity of the intestinal microbiota as well as the action of probiotics on the intestinal microbiome remain to be clarified. It is obvious that future trials should focus on clarifying the multifactorial association of the role of the cytochromes CYP (P450) in the various disease states, environmental toxic effects or chemical exposures and nutritional status. It also worth to mention once more that the effectiveness of a probiotics is strictly dependent on the strain and the dose received.

**Figure 2:**
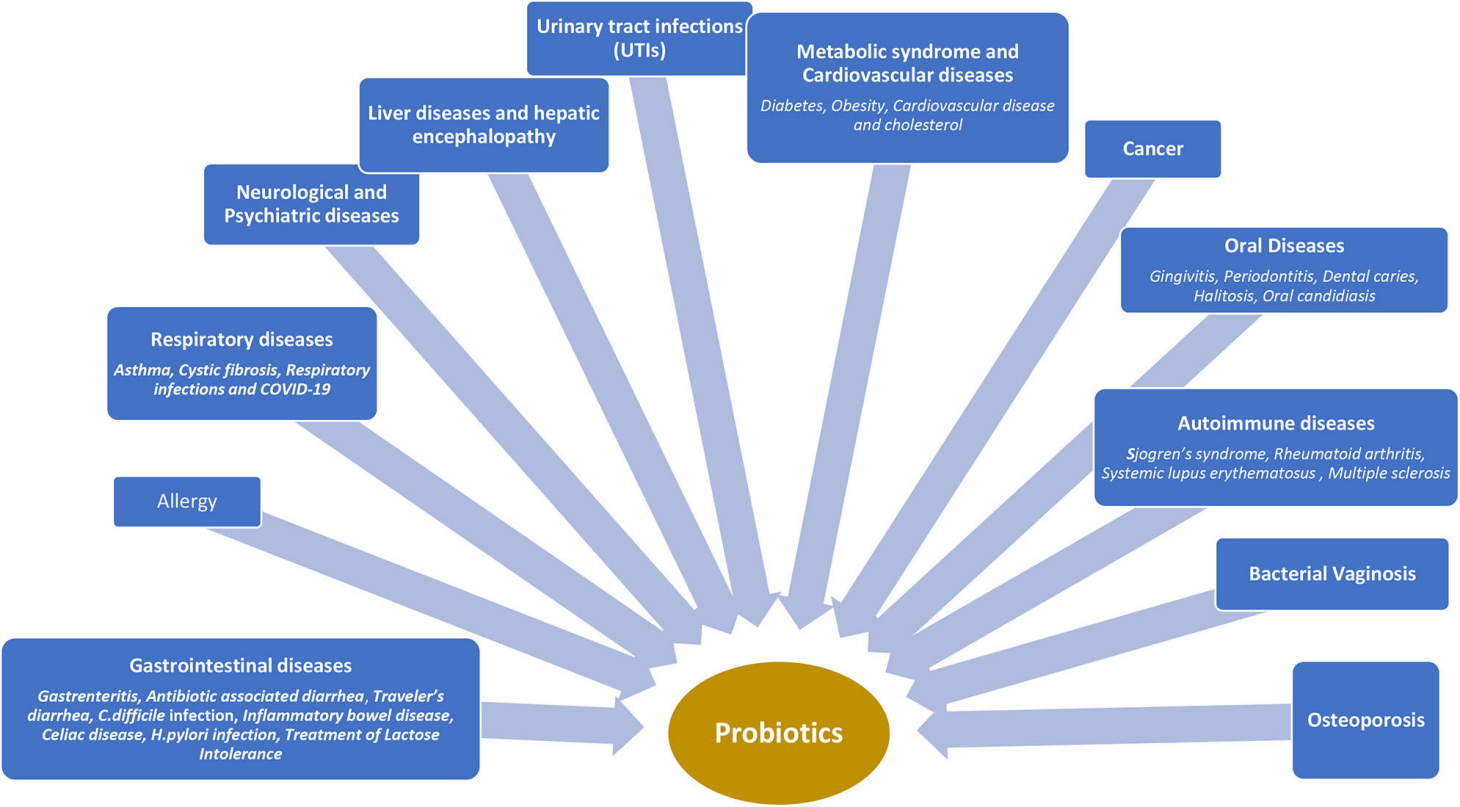
Clinical use of probiotics in different disease states.

Probiotics have a beneficial impact on the immune system by stimulating non-specific immune response, improving several disease states and alleviating allergies (Figure 2).

As it is well known, pathogens induce a pro-inflammatory response in epithelial cells by activating transcription factor activating transcription Nuclear Factor-κB (NF-kB) which keeps a crucial role in immunity, inflammation, and cell proliferation. There is evidence that probiotic strains have an effect on epithelial immune activation by blocking this factor (229).

Administration of probiotics improves the immune system in multiple ways; stimulated production of natural antibodies IgM and IgG levels systemically, increased IgA antibodies locally and systemically as well as interferon (230) and increased phagocytosis ability which modulate cytokines presence (231). In this spirit, probiotics have shown immunostimulatory effects that may be associated to the initial inflammation following human macrophages response (231).

It is emphasized that early intestinal colonization with probiotic microorganisms such as Lactobacilli and Bifidobacteria offers subsequent protection from many different types of diseases. In addition, the beneficial probiotic microflora dominated by Bifidobacteria and Lactobacilli could modify the gut microbiota by reducing the risk of cancer following their capacity to decrease β-glucoronidase and carcinogen levels (37).

Moreover, probiotics are able to produce antimicrobial substances,bacteriocins and lower the pH in order to inhibit pathogens (232), compete for nutrients with pathogens and finally enhance the intestinal barrier function (1, 224).

The integrity of the intestinal barrier is a hallmark of a healthy intestinal ecosystem (233). As discussed many factors are contributing to this issue.

A variety of *in vitro* and animal studies implied the significance of the human microbiota and the improvement of the mucosal barrier function by probiotic treatment (3, 234).

In spite of the aforementioned, there are difficulties to extrapolate results of these studies to human populations.

Multiple clinical trials have been conducted so as to evaluate the prophylactic and therapeutic effect of probiotics in different diseases and states, especially in infections after the failure of multiple courses of antibiotics.

At this point, we underpin that it is catalytic that more clinical studies should be undertaken in a large sample of diseased populations in order to evaluate the probiotics therapeutic potential. Selection criteria, efficacy and safety issues of probiotics should be considered as well as the fact that the probiotic ability seems to be strain-dependent.

In this review, we tried to summarize current knowledge on probiotics' application and therapeutic potential in different disease states and despite their possible benefits, the lack of sufficient evidence for their efficacy and safety profile makes the probiotic use a long-lasting debate.

## Author Contributions

ES focuses on all clinical aspects. All authors contributed to the article and approved the submitted version.

## Conflict of Interest

The authors declare that the research was conducted in the absence of any commercial or financial relationships that could be construed as a potential conflict of interest.
